# Certain Effects of Malaria

**Published:** 1867-11

**Authors:** Jno. C. K. Crooks

**Affiliations:** New Baltimore, Mich


### CERTAIN EFFECTS OF MALARIA.

BY. JNO. C. K. CROOKS, M. D., NEW BALTIMORE, MICH.

IT is not my purpose to write a general dissertation upon that mysterious cause of disease, *malaria*, but to call the attention of the profession to certain of its effects upon the human constitution, and more particularly upon the teeth and other organs of the mouth. Malaria, like any poisonous or antagonistic substance when taken into the system, produces disease in direct proportion to the *quantity* or the *power* of *the body to resist its presence.* Thus do we explain the fact that a residence of several weeks in our temperate climate, in a malarious district, is necessary to produce ague, while a night's exposure in the tropics will at once develop it in all its force; and, again, a healthy person may live along with apparent impunity where ague is prevalent, until some other disease has lowered his vital energies, and then it seizes him as a victim. From these principles we might infer that wherever malaria exists, there the inhabitants are more or less under its influence, whether ague presents itself or not; in other words, *that there are* CERTAIN EFFECTS of *malaria, certain* VITAL PHENOMENA, *which are directly traceable to malaria, as much so as that peculiar one which we term* INTERMITTENT FEVER, *and which are owing to a* MINIMUM DOSE of THE POISON. It is these phenomena that I shall more especially take into consideration.

In our investigation of all fevers, of diseases, the essence of which is that peculiar condition of the system variously termed "febrile action," "pyrexia," "idiopathic fever," etc., we find that the first impression of the morbid cause is upon the *nervous system.* This "conservative power," this seat of the "vis medicatrix naturæ," is alive to the presence of any poisonous influence, and early gives evidence to that effect, by setting up those wonderful processes whereby they may be eliminated or their deleterious effects neutralized. Now, these efforts of nature to preserve the system in a state of health, when the causes of disease are present, will possess an infinite variety, according (as it has been stated before) to the constitutional vigor, or the amount of poison present; and, (it may be added) according to constitutional peculiarity. If the amount is small, and the strength of the body good, the morbid effect may never go beyond the nervous system. This portion of the intricate machinery of which we are composed may be able, by great effort, to keep alive and in a healthy condition all the vital processes of the body; circulation, digestion, nutrition, secretion, elimination, etc. Again, should this nervous system *fail* to accomplish this, the morbid impressions may *extend* and some of the above functions may become disturbed, and in this manner all the phenomena of *fever* present themselves. To produce such a result, we must have, *first of all, a disturbance of the nervous or sensorial functions; secondly, derangement of the function of secretion and excretion; and lastly, derangement of the circulation.*

The nervous system suffering *first*, and that disturbance, never, in many instances, extending to other functions and thus developing that condition termed fever, we should expect, in such cases, a class of phenomena or symptoms *peculiar to that system and its known physiological actions*, and this, in every instance, holds true. The power of the nervous system being over-taxed in its labor to preserve order in the multitudinous operations of the body, or the nervous centres being themselves disturbed by the morbid cause under consideration, we have more marked, perhaps, than any other symptom, a condition of "exalted sensibility," "irritability," "nervousness," etc. The brain and spinal cord feel more keenly, than in health, every impression made upon the nerves, they are over-susceptible and this over-susceptibility works out many interesting results, which are both curious and important to the practitioner of Dentistry as well as of Medicine.

As we thus have an increased sensibility of the nerves, of course that sensibility may extend to or be present in any of the nerves of sensation and may manifest itself *generally* in an irascibility of temper, petulance, restlessness, wakefulness, etc., or it may be exhibited *specially* as in cephalalgia, hemicrania, tic doloreux, lumbago, sciatica, and even odontalgia, or a *sensitiveness of the teeth.* This latter state of things *is extremely well marked in highly malarious districts.* Let a practitioner of Dentistry go from a locality free from intermittents into one where they are the prevailing diseases, and unless previously well informed upon the subject, he will be *astonished* at the difference in the persons presenting themselves for operations. The first thing that will attract his attention will be the *timidity* of his patients. They will start at the least approach of an instrument, and in the operation of extracting teeth, where he has never failed to inspire confidence, there is an intense distrust, and from having had nothing but heroes and heroines to operate upon, he has unexpectedly discovered a race of cowards! Again, he may fill a tooth, which upon excavating the cavity, may have evinced a trifling sensitiveness, and at which he would "wink" under previous circumstances, he is now surprised with the return of his patient with the stereotyped complaint that he "can neither eat nor drink any thing hot or cold." In short, the touch of the forceps or the excavator, the file or the scaler, "wakes up" a sensitiveness which would seem to indicate, to the astonishment of the operator, that his new patrons *are endowed with nerves everywhere,* that they stand out at all points in defence of the castle they occupy, like bristling steel from a well mounted fortress!

Now, what modifications of treatment is demanded by this apparent anomaly, this pathological condition. Evidently, if the Dentist is a wise man, accustomed to analyze his cases, he will shape his proceedings according to *indications*, and operate only when he is *compelled* to operate, *until the nervous system is properly invigorated by suitable remedial agents*, by wine, bark, iron, quinine, and arsenic. He might as well expect to meet with success in filling upon an exposed nerve as to place a filling in contact with sensitive dentine, where there is that "irritability" of which I have made mention above. And it is just this state of things, in my humble opinion, which will frequently account for the different degrees of success experienced by operators of equal skill. The one has had an over-sensitive nervous system with which to contend, while the other has been blessed with healthful constitutions ready to accommodate themselves to circumstances; in the one instance, metaphorically speaking, the teeth were querelous and fault-finding, while in the other they were good-natured and obliging, ready to keep quiet under any treatment short of utter destruction.

To practice Dentistry in such a locality is something more than to practice where the hue of health is painted upon every countenance. To "fill a fang," for instance, and preserve the tooth is a stupendous undertaking, and he who counts his successful cases by the hundreds, may well congratulate himself that he is not in a malarious district.

But this *irritability* is not all. Sooner or later the nervous system *fails to preserve order* and the secretions and excretions become deranged. That large gland in the abdominal cavity, the liver, whose office it is to secrete bile, and the healthy performance of which has so much to do with a correct digestion, become disordered; it no longer does its work as it should, the digestive organs lose their tone, the salivary glands and mucous follicles of the mouth fall under the baneful influence, the gums become swollen and spongy, a tenacious and sticky secretion clings around their edges, and lastly the teeth, unless great care is taken, become coated over with an irritating tartar, get loose, and are ready to drop from the alveoli, overcome by the accumulation of evils. This is a sad picture, yet how often it is seen in "bilious" localities. In deed, so common is it, that the attention of the physician is often excited from its resemblance to the constitutional effects of mercury, and so close is this resemblance occasionally, that mercurialization *cannot be detected* until the peculiar fetor attending it is perceptible in the breath.

The PATHOLOGY of these conditions, in some respects, is very well understood, while in others there is more or less obscurity. The exact state of the nerves and nervous centres in cases of "over-sensitiveness" is but a matter of conjecture. Some pathologists consider it a "condition bordering upon or approaching inflammation," others that it *is* "neuritis, either in the track of the nerves, or at their union with the nervous centres," others, that "it is congestion," while a few consider it the "direct effects of a poison in the blood," like a narcotic, etc. Which of these theories is correct is not of much consequence so long as we know the remedy. As to the pathology of the diseases which follow *next* in the chain of morbid effects, we know much more, and can say very safely, that it is a *congestion*, more or less passive in its character, arising from a want of action in the capillaries from nervous exhaustion and from a change in the normal condition of the blood. The capillaries are *enlarged*, *they have not the power to contract upon their contents*, and this state is aggravated by the want of a *healthy* stimulus from the blood circulating through them. Thus the blood flows sluggishly from this want of tone in the capillaries, notwithstanding the pulsations of the heart may be more frequent than natural. Now, as a direct result of this torpid condition of the capillaries, the circulation becomes loaded with effete matter from the disintegration of the tissues, and instead of a healthy and life-giving fluid from which vigor is imparted to the system, we have a poisonous compound, which completely staggers the "elective affinity" of the old authors, or the "chemical affinity" of more modern times. Under such circumstances disease is inevitable, and instead of order, there will be inexplicable confusion, so that the organs everywhere, particularly those concerned in secretion and excretion fall into a serious state of derangement or disability.

The TREATMENT for this aggregation of ills must, as it has been already remarked, consist in a *general sustaining and tonic plan.* The anti-periodics, at the head of which stand the preparations of bark, are indispensable. Although the phenomena of ague may not be present, still that peculiar state of the nervous system upon which ague depends has been induced by malaria, and nothing can reach it so effectually. But, as preliminary to the administration of tonics, a *thorough alterative treatment is of the greatest consequence.* The liver, above all the secretory organs, as that will always be suffering most, must be stimulated into greater activity. Mercury, in some form, should be given in small doses, and to increase its activity and to meet other indications, it would be well to combine it with ipecac and capsicum. The capsicum imparts tone to the stomach, while the ipecac determines to the surface, thus relieving, in a measure, the congestion of internal organs and materially aiding the operation of the mercury. While the alterative is being given, a careful watch of the dejections should be enjoined on the patient, and the moment they are well tinged with bile the mercury should be discontinued. A recurrence to this treatment may be demanded during the progress of the case, still if the medicine has been given in *moderate doses* and the *secretions slowly affected,* the impression will be quite permanent and further medication for the above purpose unnecessary. As soon as the liver has responded to this treatment, it will be discovered that the other secretions are also changed for the better. The urine, which has been loaded with the lithates will have become clearer, the skin, which has been sallow and dry, will have changed to a whiter and softer texture; and the saliva, which has before been scant, tenacious and bitter will have been restored to its normal condition, and the teeth once more have a cleansing and purifying fluid in which to be bathed, instead of the thick and corroding compound which obtains under such circumstances, and which is enough to dim the lustre of the most brilliant masticators.

As soon as this change is accomplished, tonics can be given; and in addition to the preparations of bark (from the impoverished condition of the blood), iron should be freely administered. The most effectual as well as elegant combination will, perhaps, be sulphate of quinine and citrate of iron. These should be steadily and persistently given, watching in the meantime the different secretions, until the *blush of health is imparted to the faded cheeks, and the red globules restored in quantity and quality to the circulation!* To stop short of this would be to deliver our patient again to the destroyer. This must be the goal to be reached, and long exertion must be put forth to accomplish such a desirable end.

In addition to the above general measures, a well regulated local treatment will accomplish much. Early in the management of such cases, the chlorate of potash or a weakened preparation of Labaraque's solution will have a wonderful effect upon the unhealthy condition of the saliva, neutralizing many of its poisonous properties, and imparting tone to the gums, the mucous follicles, etc. These can be applied alone, or combined with tannin, tincture of kino or catechu, which, from their astringent properties, will assist a great deal in giving firmness to the loose and spongy gums.

With such treatment, wisely and perseveringly carried out, we can promise a restoration and recovery to our patients, and a condition obtained where a simple operation upon the teeth shall not be a dread both to the Dentist and his subject.

New Orleans, Oct. 7, 1867.

Messrs. Editors:

Believing it the duty of every Dentist who has the welfare of the profession at heart, to impart any new idea that may prove beneficial, I send you my method of preserving exposed pulps. Impressed for many years with the importance of saving the pulp alive, in every possible case, I have tried with varying success, every material that I have seen well recommended, for "capping the nerve," finding none of them entirely satisfactory.

We want something that will prove *congenial*, when brought in contact with the exposed pulp—that will be capable of thorough adaptation to the wall of the cavity, and that will protect the pulp, not only from the pressure of the filling, but also, from the changes of temperature.

Having about two years since an unusually difficult case, in which the patient (a lady) was very desirous of having a pulp saved, and being satisfied that nothing which had been tried, would prove equal to the occasion, it occurred to me that *vulcanized rubber*, ought to answer the conditions, better than anything that had been used.

The tooth was the right superior, central incisor, cavity very large, in the labial surface, exposure large, but pulp healthy, and patient ditto, age thirty, temperament, nervous sanguine. I took a piece of rubber plate, with one side finely polished, and after filing it to the proper thickness, cut out the cap, in form to suit the cavity, then, with a fine file, dressed the edges thin, leaving it thick enough in the central portions, to sustain the filling. Having the cavity well prepared, I placed the cap over the exposed pulp, and filled with gold.

Notwithstanding the size of the cavity, and its peculiarly exposed position to all thermal changes, the rubber being a nonconductor, and the thin and pliant edges giving it a thorough adaptation, proved so good a protection, that the patient has never felt any inconvenience, even in drinking icewater. Thus encouraged, I have tried it in, perhaps, fifty cases since, being careful that the pulp shall be always healthy, and although I have used it in each case, with the understanding that it was an experiment, the patient promising to inform me of any want of success, I have not heard of a single failure.

As my experience in this method of saving pulps has been so exceedingly satisfactory, I offer the idea to the profession, hoping that by so doing, I may enable others to *save more teeth alive*, and induce them to abandon the too common practice of murdering every dental pulp that has a slight exposure.

J. R. WALKER.

